# Convergent validity: agreement between accelerometry and the Global Physical Activity Questionnaire in college-age Saudi men

**DOI:** 10.1186/s13104-016-2242-9

**Published:** 2016-09-08

**Authors:** Shaea A. Alkahtani

**Affiliations:** Department of Exercise Physiology, College of Sport Sciences and Physical Activity, King Saud University, PO Box 1949, Riyadh, 11441 Saudi Arabia

**Keywords:** Global Physical Activity Questionnaire, Saudi men, Sedentary behavior, Self-reported physical activity

## Abstract

**Background:**

The Global Physical Activity Questionnaire (GPAQ) has been recommended for the international tracking of physical activity (PA). This study aimed to investigate the agreement between the GPAQ and accelerometry, as well as the test–retest reliability of the GPAQ in Saudi college-age men.

**Methods:**

The participants included 62 male students, aged 20.0 ± 1.1 year, with a mean body mass index (BMI) of 24.1 ± 6.3 kg/m^2^. This study used a cross-sectional comparison of measures design. Participants completed the GPAQ twice (2 weeks apart) and wore accelerometers for seven consecutive days.

**Results:**

The agreement between the GPAQ and accelerometry for moderate to vigorous PA (MVPA) was weak (r ≤ 0.32). Participants underreported sedentary time relative to accelerometer measurements (∆ = 3.4 h/day). BMI was statistically associated with increased bias between the two methods. However, correlations between the GPAQ test and retest for MVPA and sedentary time were moderate to strong (r = 0.44–0.78).

**Conclusion:**

The GPAQ is reliable, but had low agreement with accelerometry for estimating MVPA, and very low agreement with accelerometry for estimating sedentary time in college-age Saudi men. Individual participant characteristics should be considered when using the GPAQ to estimate sedentary time. Adapting the current GPAQ to build a regional PA questionnaire is recommended.

**Electronic supplementary material:**

The online version of this article (doi:10.1186/s13104-016-2242-9) contains supplementary material, which is available to authorized users.

## Background

There is evidence for the importance of physical activity (PA) to human health, but measuring PA levels in population surveillance is challenging. For example, age- and sex-based PA patterns are similar between objective and subjective measures, but self-reported PA substantially overestimates accelerometer-measured PA [1]. This lack of agreement between self-reported and objective measure of PA increases the probability of misinterpretation of epidemiological research, which could lead to improper public health program design. Thus, it is important to establish the most appropriate PA measurements for use in health programs, research, and surveillance at the international, national, and local levels [2].

The World Health Organization (WHO) developed the Global Physical Activity Questionnaire (GPAQ) to estimate PA, which is especially relevant in developing countries. For this purpose, a WHO expert working group on PA measurement provided a revised draft of the GPAQ for global evaluation. The draft questionnaire was completed by 2657 men and women from nine developing countries and showed moderate to strong reliability, moderate to strong positive correlation with the International Physical Activity Questionnaire (IPAQ), and poor to fair correlation with criterion validity [3]. Approximately 50 developing countries, including Saudi Arabia, currently use the GPAQ version 2, and it is now recommended as a suitable PA surveillance instrument for developing countries [4]. However, inconsistencies in the validity of the GPAQ have been observed in several recent studies. For example, the GPAQ validly measured moderate to vigorous PA (MVPA), but was a less valid measure of sedentary behavior, in Northern Irish adults [5]. Validity of the GPAQ was examined against direct (accelerometer) and indirect (physical fitness and body composition) criterion measures among American adults, and the results showed that reported MVPA was inversely related to percent body fat and waist circumference, and positively related to VO_2max_, and measured MVPA, although increasing bias with higher levels of overreporting of PA was observed [6].

The Gulf Cooperation Council (GCC) consists of six countries, including Saudi Arabia. Most studies in the region have used self-reported PA surveys and various daily life domains to assess PA levels and the associations between PA level and various health aspects. For example, in one study of Omani adults that used the GPAQ to assess PA domain, the authors pointed out that the validity and reliability testing of this instrument did not include populations from the Arab world [7]. That study found a lower risk of developing the metabolic syndrome in those with higher work and transportation, but not leisure time, activity; the authors also provided recommendations for national PA guidelines, policies, and programs. In Saudi Arabia, the GPAQ has also been used to assess whether adults met the PA recommendations for cancer prevention [8]. Furthermore, another study in Saudi Arabia used a pedometer and diary-based daily step count method to record daily PA, and found an association between PA and health beliefs among Saudi women [9]. Another PA questionnaire that had been validated against a pedometer has been used among Saudi adolescents [10]. The Arabic short form of the IPAQ has also been validated, but this version is limited to a telephone interview. Most of these studies used more than one instrument to minimize errors because, while pedometers can be used for promoting PA, they are not recommended for use in assessing exercise volume [11].

While the validity of self-reported PA measures has been examined against accelerometers in the scientific literature, the evaluation of self-reported PA methods is currently being reconsidered. Kelly et al. [12] raised the point that test validity combines face, content, agreement (convergent or criterion), and time (concurrent or predictive) assessments. They concluded that concurrent measures of self-reported PA and accelerometry do not mean the accelerometer is a criterion because the accelerometer does not cover all of the domains of the self-reported PA questionnaire; thus, there is a lack of content validity [12]. Questionnaires are developed to capture frequent behaviors in specific contexts such as occupation, household, and transportation (e.g., walking and bicycle) activity. While accelerometers might have strong content and concurrent agreement with some recreational activities in terms of duration, frequency, and intensity, they do not have the same level of agreement with other sports, such as swimming, cycling, and resistance training. Thus, Kelly et al. [12] reemphasized that the PA measurement hierarchy is incorrect because it is based only on total energy expenditure; energy expenditure should not be the sole PA measurement criterion, and different assessments have different strengths and weaknesses. Troiano et al. [13] recently stated that the relationships between self-reported and accelerometry-based PA are often of low to moderate strength because these two measures are not equivalent, and therefore are not interchangeable.

The evaluations of PA and sedentary behavior in relation to health aspects are complex, and some authors recommend a PA framework, such as Medical Research Council Diet and Physical Activity Assessment Toolkit [12], or a specific community-based self-reported PA questionnaire, such as the Physical Activity for Adults Questionnaire (PAAQ) [14]. Thus, it is important to provide a framework of PA in the GCC countries, including Saudi Arabia, using global and specific self-reported PA as well as objective measures (e.g., triaxial accelerometry). The current study is the first to attempt such a study, and aims to evaluate the agreement between the existing global questionnaire (GPAQ) and accelerometry among Saudi young men.

## Methods

### Participants

Participants were recruited from the Preparatory Year at the University of Dammam in Saudi Arabia through e-mails and posted notices. All Preparatory Year students were eligible to participate in the study, and the goal sample size was 10 % of Preparatory Year students. Using the G*Power 3.1 calculator for sample size based on an effect size of f = 0.40, an α = 0.05, three groups, and two degrees of freedom, we calculated that a sample size of 61 would achieve 80 % power (1 − β).

### Study design and setting

This study employed a cross-sectional comparison of measures design. It was conducted at the University of Dammam. All subjects provided written informed consent prior to participation in the study. Participants came to the Training Hall of the Preparatory Year College, where height (to the nearest 0.5 cm) and weight (to the nearest 0.1 kg) were measured using a digital scale fitted with a height column (Gima, Gessate, Italy). Thereafter, PA was measured using the questionnaire and accelerometers.

### Data measurement

The official Arabic version of the GPAQ (http://www.who.int/chp/steps/GPAQ/en/), along with the official show cards (containing adapted photos and Arabic translations of headings and examples) were used in the current study. The GPAQ has four domains: moderate and vigorous activity at work, travel to and from places, moderate and vigorous recreational activities, and sedentary behavior. As all study participants were students who do not engage in any moderate or vigorous work activities, the work domain was excluded from the final analysis. To examine test–retest reliability, the GPAQ was administered twice, first at the initial visit before wearing the accelerometer and then 2 weeks later. The questionnaires were self-administered in the presence of research assistants who assisted and answered questions.

PA was measured using accelerometers (ActiGraph-wGT3X-BT, Pensacola, FL, USA) for seven consecutive days. All participants were instructed to wear the device on their right hip throughout the day except for while sleeping or in contact with water. Dependent variables obtained from the accelerometers were computed using ActiLife software (ActiLife, v 6.11.6., 2009, ActiGraph, Pensacola, FL, USA). Wear-time validation was computed using the Troiano algorithm [15]. A non-wear period was defined as 60 consecutive minutes of zero magnitude counts, with an allowance of two consecutive minutes of non-zero counts less than 100 counts/min. A minimum wear-time of 10 h/day for 4 day was applied to define a valid day. Vector magnitude thresholds were divided into five categories according to the Freedson cut points for adults, as follows: sedentary was 0–99 counts/min, light was 100–1951 counts/min, moderate was 1952–5724 counts/min, vigorous was 5725–9498 counts/min, and very vigorous was 9499 counts/min and above [16]. Furthermore, time in light activity in a minimum of 10-min bouts was calculated using ActiLife software. Light PA (LPA) in a minimum of 10-min bouts was calculated per day and multiplied by 7 to compute the total light activity in a minimum of 10-min bouts per week. Differences between the GPAQ-reported and accelerometer-measured activity levels were calculated as ∆ = [(accelerometer-measured) − (GPAQ-reported)].

### Statistical methods

Data were analyzed using SPSS (version 22 Chicago, IL, USA). Continuous data are presented as mean ± standard deviation (SD) and median (1st and 3rd quartile) for variables with Gaussian and non-Gaussian distributions, respectively. Categorical data are presented as frequencies and percentages (%). All continuous variables were tested for normality using the Kolmogorov–Smirnov test and tests for skewness and kurtosis. If the Kolmogorov p value was significant or the skewness or kurtosis value was less than ±1 or ±2, respectively, the data were considered non-Gaussian and non-parametric analyses were used to analyze the data. PA level- and BMI-based differences between participants’ ∆ MVPA and ∆ sedentary time were tested with the Kruskal–Wallis test for non-Gaussian distributions. Correlation coefficients between variables were calculated using Spearman’s correlation analysis. A p value of <0.05 was considered statistically significant.

## Results

A total of 96 out of approximately 700 male students in the Preparatory Year volunteered to participate in the current study. All participants completed the measurements, but only 62 achieved the required accelerometer daily wear time (10 h/day of wear-time for a minimum of 4 days, including 1 weekend day); the average wear-time was 14.3 ± 1.5 h/day for an average of 5.58 ± 1.2 day/week. The final sample was 20.0 ± 1.1 year old, with a body mass index (BMI) of 24.1 ± 6.3 kg/m^2^. Table 1 shows the study population and the included participant sample size and descriptive data.

**Table 1:** Population and participant descriptive data

Variable	Value
Study population (N)	700
Study participants (n_1_)	96
Accelerometer wear-time inclusion criterion	4 days, 10 h/day
Average accelerometer wear days (d)	5.58 ± 1.2
Average accelerometer wear time per day (h)	14.3 ± 1.5
Participants included in analysis (n_2_)	62
Age (year)	20 ± 1.1
BMI (kg/m^2^)	24.1 ± 6.3

Data expressed as mean ± SD

*N* population sample size, *n* study sample size, *BMI* body mass index

The GPAQ test–retest correlations are shown in Table 2. The r values were strong for all variables, except for the correlation of moderate exercise level, which was moderate. It should be noted that the 1st quartile of all PA patterns indicates that 25 % of participants scored zero; these scores were consistent in the retest. Vigorous PA had the strongest test–retest correlation.

**Table 2:** Test-retest reliability of the Global Physical Activity Questionnaire

Variable	Test 1	Test 2	r value
Active commuting (min/day)	20.0 (0–60.0)	30.0 (0–60.0)	0.69 **
Active commuting (min/week)	52.5 (0–236.3)	90.0 (0–270.0)	0.77 **
Vigorous exercise (min/week)	90.0 (0–232.5)	75.0 (0–180.0)	0.78 **
Moderate exercise (min/week)	60.0 (0–178.8)	60.0 (0–180.0)	0.44 **
Sedentary time (h/day)	6.0 (3.1–9.0)	6.0 (3.0–9.0)	0.70 **

Data expressed as median (1st–3rd quartiles) and Spearman correlation coefficients

** Significant correlation at p ≤ 0.01

The agreement between the GPAQ and the accelerometer is shown in Table 3; the r value of the correlation between the GPAQ and the accelerometer was low for MVPA and very low for sedentary behavior. While the 1st quartile of moderate PA using the GPAQ was 0, it was 128 min/week for the accelerometer. In addition, the range of sedentary time values was larger for the GPAQ than for the accelerometer.

**Table 3:** Global Physical Activity Questionnaire and accelerometer agreement for reported moderate to vigorous activity and sedentary time

Variable	Accelerometer	GPAQ	r value
Moderate PA (min/week)	188.0 (128.0–289.0)	60.0 (0–180.0)	0.24 (p = 0.058)
Vigorous PA (min/week)	3.0 (0–9.0)	75.0 (0–180)	0.32 **
MVPA (min/week)	273.5 (178.6–405.2)	167.5 (60.0–345.0)	0.32 *
Sedentary time (h/day)	9.4 (8.6–10.2)	6.0 (3.0–9.0)	0.08

Data expressed as median (1^st^–3^rd^ quartiles) and Spearman correlation coefficients

*GPAQ* Global Physical Activity Questionnaire, *MVPA* moderate-vigorous physical activity, *PA* physical activity

* Significant correlation at p ≤ 0.05

** Significant correlation at p ≤ 0.01

**Fig. 1:**
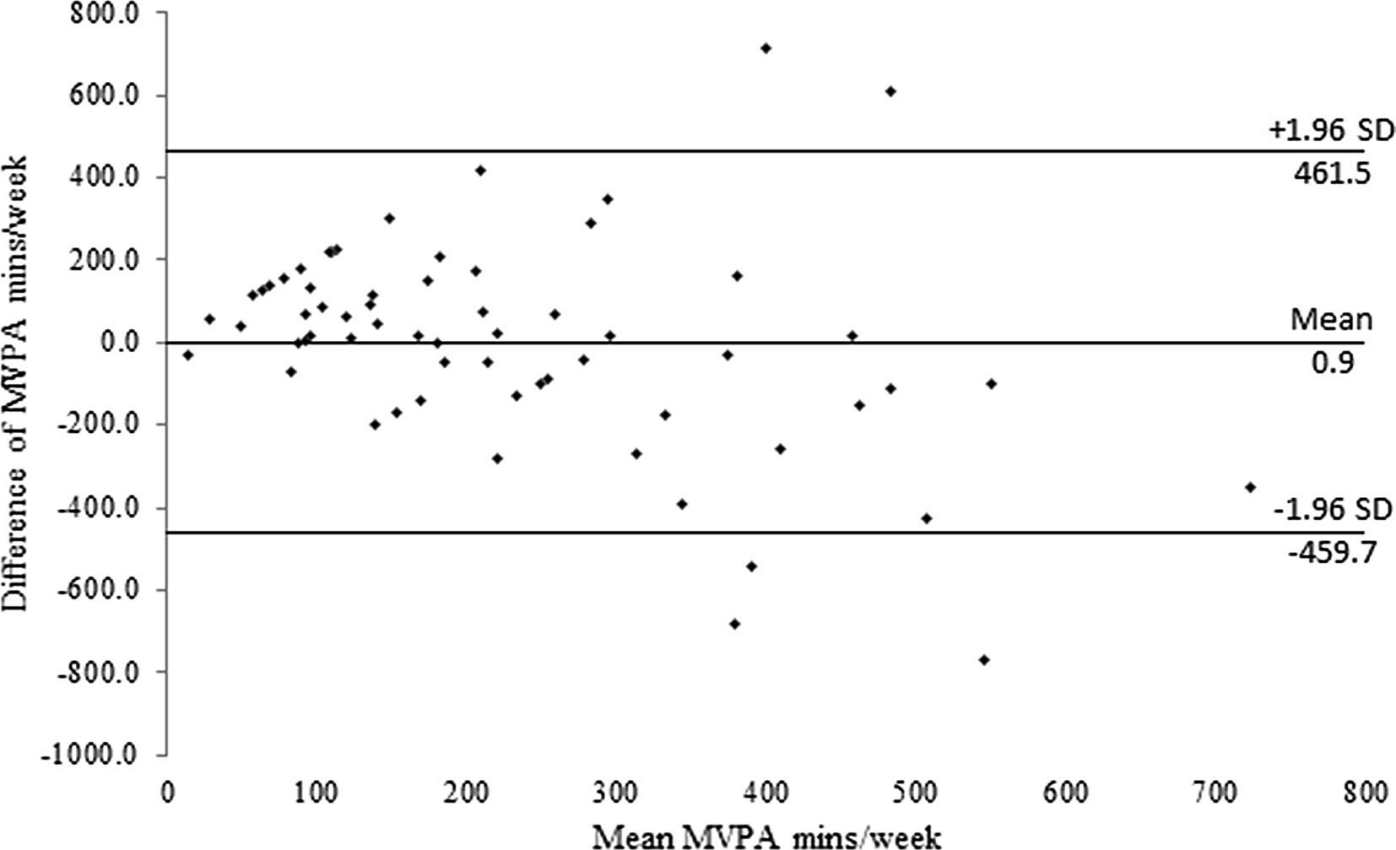
Bland–Altman plot of the difference versus mean of moderate to vigorous physical activity level. Difference of moderate to vigorous physical activity (MVPA) was calculated as [(accelerometer-measured MVPA) − (questionnaire-reported MVPA)]. The upper and lower limits of agreement (±1.96 SD) are indicated

**Fig. 2:**
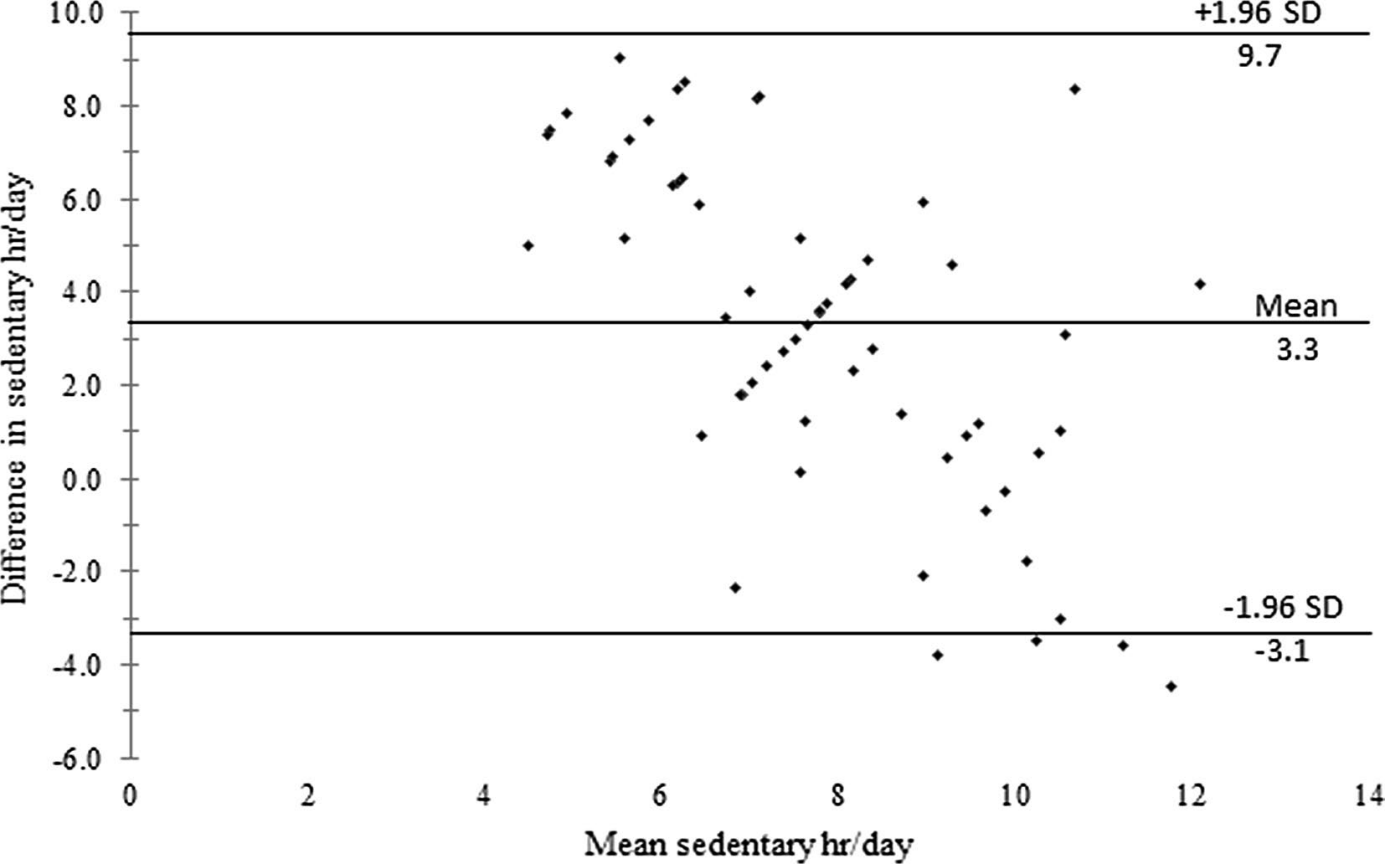
Bland–Altman plot of the difference versus mean of sedentary time. Difference of sedentary time was calculated as [(accelerometer-measured sedentary time) − (questionnaire-reported sedentary time)]. The upper and lower limits of agreement (±1.96 SD) are indicated

Bland–Altman plots comparing the difference and mean GPAQ and accelerometer results for MVPA and sedentary time are shown in Figs. 1 and 2. The upper and lower limits of agreement (±1.96 SD) are shown, illustrating that some participants were outside the limit of agreement; these represent low agreement between the GPAQ and the accelerometer.

Table 4 shows the differences between the accelerometer and the GPAQ measurements of MVPA and sedentary time based on PA level. PA level was divided into three groups as follows: very active, ≥250 min MVPA/week; active, 150–249 min MVPA/week; and inactive <150 min MVPA/week. It should be noted that most study participants were active, with 85 % of the participants engaging in 150 or more min/week of MVPA. The differences between the GPAQ and the accelerometer estimations of sedentary time approached significance (p = 0.052), and the underestimation of sedentary time increased with increasing levels of PA. While this was not clear based on the median values, it was evident when examining the 1st and 3rd quartiles.

**Table 4:** Differences between accelerometer and the Global Physical Activity Questionnaire measurements based on physical activity level

Variable	Very active (n = 32)	Active (n = 20)	Non-active (n = 9)	p value
∆ MVPA (min/week)	94.5 (−130.5 to 292.0)	−12.5 (−129.5 to 89.0)	−74.0 (−471.0 to 61.0)	0.840
∆ Sedentary time (min/day)	205.2 (89.5–364.4)	157.7 (48.8–310.9)	201.6 (−93.0 to 436.0)	0.052

*∆* data measured using accelerometer-data reported using GPAQ. Data expressed as median (1st–3rd quartiles)

*GPAQ* Global Physical Activity Questionnaire, *MVPA* moderate-vigorous physical activity, *Very active* ≥250 min MVPA/week, *Active* 150–249 min MVPA/week, *non-active* <150 min MVPA/week

Table 5 shows the differences between the accelerometer and the GPAQ for MVPA and sedentary time based on BMI. Participants were divided into four BMI groups, as follows: underweight (BMI <18.5 kg/m^2^), normal weight (BMI 18.5–24.9 kg/m^2^), overweight (BMI 25.0–29.9 kg/m^2^), and obese (BMI ≥30.0 kg/m^2^). Based on BMI, 30 % of participants were overweight or obese. A significant difference was observed for ∆ sedentary time (p ≤ 0.05) between the obese and underweight groups. Agreement between the accelerometer and the GPAQ decreased with increasing levels of BMI.

**Table 5:** Differences between accelerometer and the Global Physical Activity Questionnaire measurements based on body mass index

Variables	Underweight (n = 17)	Normal weight (n = 26)	Overweight (n = 9)	Obese (n = 9)	p value
∆ MVPA (min/week)	9.0 (−74.0 to 220.0)	59.0 (−161.0 to 149.0)	68.0 (26.0–152.0)	−121.0 (−412.0 to 2.0)	0.21
∆ Sedentary time (min/day)	98.5 (14.0–171.7)	224.2 (49.5–383.4)	278.3 (161.0–313.6)	313.4 (201.6–388.0)^a^	0.03

*∆* data measured using accelerometer–data reported using the GPAQ

Underweight (BMI <18.5 kg/m^2^), Normal weight (BMI 18.5–24.9 kg/m^2^), Overweight (BMI 25.0–29.9 kg/m^2^), Obese (BMI ≥30.0 kg/m^2^)

Data presented as median (1st–3rd quartiles)

*GPAQ* Global Physical Activity Questionnaire, *BMI* body mass index, *MVPA* moderate-vigorous physical activity

^a^Indicates a significant difference from Underweight, p ≤ 0.05

Further comparison of commuting activity using the GPAQ and LPA with 10-min minimum accelerometer bouts revealed that the median values of commuting activity and LPA with 10-min minimum bouts were 679.9 (521.7–885.8) and 90.0 (0–300.0) min/week, respectively. Minimum and maximum values were 270 and 2042 min/week for commuting and 0 and 2100 min/week for LPA. The median time of 10-min minimum bouts was 13.2 (12.6–14.2) min/bout, and the median number of 10-min minimum bouts per week was 36.0 (28.0–53.8); however, these variables could not be compared to the GPAQ as they are not specifically queried in the GPAQ.

## Discussion

The aims of the current study were to examine the reliability of the GPAQ using a test–retest method and to test its convergent validity through a comparison with accelerometer data in young Saudi men. Our results showed moderate to strong test–retest correlations, low correlations between the GPAQ and the accelerometer for MVPA, and very low correlations between the GPAQ and the accelerometer for sedentary time. The differences between the GPAQ and the accelerometer were consistently biased with increasing levels of PA and obesity.

The current study found a low correlation between GPAQ and accelerometry for MVPA (r = 0.32), which was slightly lower than reported in a recent study [5] that found moderate agreement between the GPAQ and accelerometry data for MVPA (r = 0.48) among middle-aged adults. Another study of 54 adults (43.1 ± 11.4 years) was in agreement with the present finding, showing a weak correlation (r = 0.28) for MVPA and a moderate correlation (r = 0.48) for vigorous PA between the GPAQ and accelerometry [6]. That study also reported that short-term (10-days) test–retest reliability ranged from 0.83 to 0.96, while long-term reliability (3 months) ranged from 0.53 to 0.83. Another study found a moderate correlation between the GPAQ and accelerometry for MVPA (r = 0.46) and strong test–retest correlation for GPAQ (r = 63) [17]. Similarly to our finding, the most sensitive measure for GPAQ based on the correlation with the accelerometer among Latinas was leisure time vigorous PA (r = 0.40) [18]. The stronger correlation of vigorous exercise compared with other PA levels might be attributed to the perception of intensity. It has been reported that responders understand intensity as emotional, rather than as physical effort, and there is a difference between respondents’ and researchers’ interpretations of intensity [19]. Individuals overestimate moderate and vigorous PA effort relative to their measured heart rate during said activity [20]. Similarly, the current study participants overestimated moderate activities, as shown in Table 3. This could be attributed to time spent playing soccer, because soccer was depicted as a vigorous activity in the GPAQ show cards, while most participants were involved in recreational soccer that was likely moderate in intensity.

A very low correlation between the GPAQ and accelerometry was observed for sedentary time in the current study, which was in agreement with the study of Cleland et al. [5]. Underestimation of sedentary behavior using the GPAQ (1130 min/week) compared with the accelerometer (3365 min/week) and a lack of correlation between the two measures has also been reported [18]. Low agreement between accelerometry and questionnaire responses was also found with the IPAQ in a large sample of men and women; participants reported 131 min/day less sedentary time compared with accelerometer data [21]. There are many possible explanations for the differences between GPAQ- and accelerometer-measured sedentary time. First, accelerometers record all activities below than 100 counts/min, including sitting and standing, as sedentary, whereas the GPAQ asks only one question regarding the total sedentary hours per day, except standing time. Some accelerometers measure breaks in sedentary time, which are designed to capture transitions from sedentary time to activity using 100 counts/min as threshold; however, this has low agreement with posture-derived breaks using a posture sensor [22]. It should be noted that, although most studies found underestimation of self-reported sedentary time, one study observed an overestimation of sedentary time compared with accelerometry data among adults [23]; and this could be because of remembering/forgotten treats. Thus, several context-specific sedentary behaviors have been examined [24], and 24-h-recall of sedentary time rather week-recall was recommended [25] for college-age individuals [26]. Lastly, while the average wear-time in the current study was 14.3 h/day, the minimum wear-time criterion was only 10 h/day. Some studies have found data based on 10 h/day of wear-time led to missing 30 % of sedentary time compared to data based on 14 h/day of wear-time; longer accelerometer wear-time (i.e., >13 h/day) was recommended for accurate estimates of daily sedentary time [27]. In the current study, participants underestimated their self-reported sedentary time by 204 min/day. Several factors related to instruments partially explain the lack of agreement between the GPAQ and accelerometer methods in estimating sedentary time; splitting the GPAQ sedentary time question into several questions throughout all of the activity domains might be a more appropriate strategy and should be examined.

The Bland–Altman plot in Fig. 1 shows that when the mean MVPA value was above the average (230 min), several participants were outside the limits of agreement. These participants increased the range of the agreement limits for the difference between the two methods. Good agreement between the two PA measurement methods should be ±90 min/week (i.e., the recommended PA level for health is ≥150 min/week and for weight loss is ≥250 min/week, with sedentary time ≤60 min/week). The Bland–Altman plot in Fig. 2 demonstrates a systematic bias in the difference between the GPAQ and the accelerometer for sedentary time. Further analysis was performed to examine the influence of indirect criterion measures (e.g., anthropometry and PA level) on agreement between the two methods. It is important to explore secondary factors that may affect the agreement between the GPAQ and accelerometry because the GPAQ is used in multiple age [28] and obesity [29] groups. For example, graded differences across categories have been observed for the GPAQ based on step count, BMI, waist circumference, percent fat, fitness, and accelerometer-measured activity [6].

The median values in the current study revealed systematic underestimation of GPAQ-measured sedentary time with increasing PA levels (Table 3). No differences between participants for MVPA based on PA levels were observed, but the Bland–Altman plot showed increased variation among some participants. A previous study found a bias toward overestimating MVPA in participants with higher levels of MVPA [17]. Alternatively, other studies have found participants with lower fitness levels overestimated their moderate PA [21]. The current participants of our study are educated young men. Educated people (holding a degree above high school) are more thorough in their PA level estimation [21]. Age also affects the understanding and interpretation of self-reported PA questionnaires [30, 31]. The educational level and age of the current participants could have improved the precision of reported PA. Similar systematic underestimation of sedentary time has been observed with increasing degrees of obesity; the difference between obese and underweight participants was significant in the present study (Table 4). Perceptual fatigue should be enhanced during body effort to further extent than during sedentary time. However, a recent study suggested that obesity did not affect the correlation between self-reported and objective PA measures [21]. The cause of the systematic underestimation of sedentary time with increasing obesity occurred, but considering the prevalence of overweight/obese participants (9 per subgroup), this requires further investigation with sufficient sample size.

One of the limitations of the GPAQ is that there is no explicit method to examine the validity of LPA lasting more than 10 min/bout. In the present study, no subjects reported bicycle transportation, so we compared active transportation with accelerometer-based LPA, assuming that active commuting was primarily casual walking requiring less than 3 metabolic equivalents (METs). Active commuting can also include brisk or uphill walking, requiring 3–6 METs. If active commuting using the GPAQ was captured as LPA and MPA using an accelerometer, time spent in active commuting using GPAQ must be greater than accelerometer-measured LPA. However, the opposite was observed, indicating an underestimation of active commuting PA levels sustained for more than 10 min. The current GPAQ estimates only time spent in active commuting; inclusion of the housework domain and LPA at work might improve the capture of LPA. Most previous studies have reported comparisons for only sedentary time and MVPA, but LPA is an important form of PA that is associated with health aspects. For example, we recently found that LPA is associated with elevated high-density lipoprotein [32]. Thus, the transportation section in the questionnaire should be expanded to include other elements such as distance, standing, and time distribution, in order to better capture all daily LPA.

### Strengths, limitations, and future directions

The strengths of this study include the fact that it may be the first to examine the agreement between the GPAQ and accelerometry in GCC countries. PA patterns can differ between different countries, and this is also affected by the age of the subgroup [33]. Therefore, data from the current study will help build an appropriate regional questionnaire based on the current GPAQ, such that the GPAQ could have different regional versions with specific questions relevant to the regional lifestyle. For example, physically active occupations and bicycle riding are not currently common among Saudis. An example of such a questionnaire is the European Health Interview Survey-Physical Activity Questionnaire (EHIS-PAQ) [34]. The limitations of current study include that it did not show how different accelerometry cut points might affect the agreement between subjective and objective methods of PA assessment, particularly as they relate to sedentary time. The outcomes of the current study should be interpreted with caution due to the small sample size. For example, though it has been previously shown, we did not find an effect of PA levels on the estimation of MVPA; this could be due to low statistical power related to the sample size. Further studies are recommended to examine the agreement between self-reported PA questionnaires and objective PA assessment methods among different subgroups, including females and children, to better understand the influence of secondary factors on individual responses. Additional comparisons between college-age students and similarly aged non-students are also suggested.

## Conclusions

In conclusion, the GPAQ demonstrated strong reliability, but weak agreement with accelerometry for measuring MVPA, and very weak agreement with accelerometry for measuring sedentary time in Saudi young men. The bias in the relationship between these two methods for sedentary behavior might be partially attributed to both instrument-based factors and individual-based factors. Indirect criterion measures of individual characteristics should be considered when using different methods of PA assessment. Further, adapting the current GPAQ to create a regional questionnaire is suggested.

## Additional file

10.1186/s13104-016-2242-9 Raw data of accelerometers and the Global Physical Activity Questionnaire in week 1 and 2, including sedentary, low, moderate and vigorous physical activity (n = 62).

## Abbreviations

BMI: body mass index
GCC: Gulf Cooperation Council
GPAQ: Global Physical Activity Questionnaire
IPAQ: International Physical Activity Questionnaire
LPA: light physical activity
MET: metabolic equivalent
MVPA: moderate to vigorous physical activity
PA: physical activity
VO_2max_: maximal oxygen consumption
